# (De)stabilization of Alpha-Synuclein Fibrillary Aggregation by Charged and Uncharged Surfactants

**DOI:** 10.3390/ijms222212509

**Published:** 2021-11-19

**Authors:** Joana Angélica Loureiro, Stéphanie Andrade, Lies Goderis, Ruben Gomez-Gutierrez, Claudio Soto, Rodrigo Morales, Maria Carmo Pereira

**Affiliations:** 1 LEPABE, Department of Chemical Engineering, Faculty of Engineering of the University of Porto, 4200-465 Porto, Portugal; stephanie@fe.up.pt; 2 Faculty of Pharmaceutical Sciences, Ghent University, Sint-Pietersnieuwstraat 25, B-9000 Ghent, Belgium; Lies.Goderis@UGent.be; 3 Department of Neurology, The University of Texas Health Science Centre at Houston, Houston, TX 77030, USA; ruben.gomezgutierrez@bcm.edu (R.G.-G.); Claudio.Soto@uth.tmc.edu (C.S.); Rodrigo.MoralesLoyola@uth.tmc.edu (R.M.); 4 Department of Cell Biology, University of Malaga, 29071 Malaga, Spain; 5 CIBQA, Universidad Bernardo O’Higgins, Santiago 1497, Chile

**Keywords:** α-synuclein, protein aggregation, Parkinson’s disease, protein secondary structure, aggregation mechanisms

## Abstract

Parkinson’s disease (PD) is the second most common neurodegenerative disorder. An important hallmark of PD involves the pathological aggregation of proteins in structures known as Lewy bodies. The major component of these proteinaceous inclusions is alpha (α)-synuclein. In different conditions, α-synuclein can assume conformations rich in either α-helix or β-sheets. The mechanisms of α-synuclein misfolding, aggregation, and fibrillation remain unknown, but it is thought that β-sheet conformation of α-synuclein is responsible for its associated toxic mechanisms. To gain fundamental insights into the process of α-synuclein misfolding and aggregation, the secondary structure of this protein in the presence of charged and non-charged surfactant solutions was characterized. The selected surfactants were (anionic) sodium dodecyl sulphate (SDS), (cationic) cetyltrimethylammonium chloride (CTAC), and (uncharged) octyl β-D-glucopyranoside (OG). The effect of surfactants in α-synuclein misfolding was assessed by ultra-structural analyses, in vitro aggregation assays, and secondary structure analyses. The α-synuclein aggregation in the presence of negatively charged SDS suggests that SDS-monomer complexes stimulate the aggregation process. A reduction in the electrostatic repulsion between N- and C-terminal and in the hydrophobic interactions between the NAC (non-amyloid beta component) region and the C-terminal seems to be important to undergo aggregation. Fourier transform infrared spectroscopy (FTIR) measurements show that β-sheet structures comprise the assembly of the fibrils.

## 1. Introduction

Parkinson’s disease (PD) is the second most common neurodegenerative disorder, after Alzheimer’s disease. Four million individuals worldwide are affected by PD, especially middle-aged men [1,2].

α-synuclein is a presynaptic neuronal protein that may contribute to PD pathogenesis [3,4]. The normal function of α-synuclein is poorly understood. It has been described that α-synuclein is involved in many synaptic processes [5]. This protein also participates in the trafficking of synaptic vesicles and in the regulation of vesicle exocytosis. α-synuclein can act like a chaperone and controls protein degradation and the assembly and distribution of the SNARE (soluble N-ethylmaleimide-sensitive fusion protein attachment protein receptors)–protein complexes, which is directly associated with the release of neurotransmitters, including dopamine [6,7]. Other attributed functions of α-synuclein include fatty acid binding, physiological regulation of several enzymes, and neuronal survival [8].

To exert these functions, α-synuclein requires proper folding [9]. However, under certain circumstances, α-synuclein misfolds and aggregates in disease-associated structures. The entire process of α-synuclein aggregation is still unclear. α-synuclein is a typical intrinsically disordered protein (IDP). Under different conditions, and in the presence of different cofactors, this protein can adopt a quantity of different conformational states. As the IDPs enclose preformed binding elements, they could be involved in a set of non-native intramolecular interactions. So, α-synuclein can convert directly into nucleus, or may form transient oligomers (highly disordered or partially structured) that eventually grow into fibrils [10,11]. These misfolded (disease-associated) aggregates are characterized by β-sheet structure [12].

Studying the conditions that favour α-synuclein aggregation is fundamental to design strategies aiming to prevent neuronal death. Stabilizing and destabilizing protein structure in the presence of surfactants can elucidate aggregation mechanisms [13]. The interaction mechanism between surfactants and α-synuclein may be used for this purpose as it has been previously suggested for the case of globular proteins and peptides [14,15,16,17,18,19,20,21,22,23]. The aim of this study was to analyse the interactions between α-synuclein and charged and uncharged surfactants in the context of protein misfolding and aggregation. These interaction studies are crucial to understand whether intracellular charged or uncharged species and surfaces favour the misfolding of this protein. As α-synuclein consists of many charged amino acids (a.a.), binding of this protein to charged surfactants could alter its spatial configuration. Moreover, hydrophobic a.a. are present in the α-synuclein sequence, which can undergo hydrophobic interactions with the surfactant tails.

In this work, the anionic sodium dodecyl sulphate (SDS) and the cationic cetyltrimethylammonium chloride (CTAC) were used as charged surfactants. In addition, octyl β-D-glucopyranoside (OG) was selected to test the effect of uncharged surfactants in α-synuclein protein misfolding. Concentrations below and above their critical micelle concentration (CMC) were used to understand the dominant interactions of α-synuclein with surfactant monomers and micelles, respectively. CMC of the surfactants was determined in the absence and presence of α-synuclein by measuring the surface tension using the hanging drop method [24]. Then, samples were characterized by ultra-structural analysis of the aggregates, and their aggregation kinetic was studied by an in vitro aggregation assay. In addition, α-synuclein secondary structure was analysed by Fourier transform infrared spectroscopy (FTIR).

The thorough characterization of α-synuclein-surfactants presented in this study will allow us to increase the chances of finding promising drugs that may ultimately lead the way to prevent the misfolding and aggregation of this protein.

## 2. Results and Discussion

**Figure 1:**
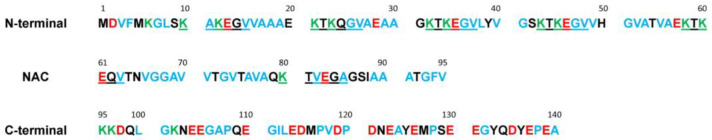
Primary structure with the amino acid (a.a.) sequence of α-synuclein. Positively and negatively charged amino acids are indicated in green and red, respectively. Hydrophobic amino acids are indicated in blue. The imperfect repeats are underlined.

The interactions between proteins and surfactants are characterized by electrostatic and hydrophobic interactions. The primary structure of α-synuclein has a sequence of 140 a.a. (Figure 1). The N-terminal region of this protein is mostly positively charged, while the C-terminal end is negatively charged (Table 1). The latter is rich in acidic residues and is responsible for the disordered structure of the protein [25,26]. This region also plays a role in α-synuclein fibrillation and subsequent aggregation [27]. The central part of the protein is the most hydrophobic domain (61–95 a.a.). Because of this region, α-synuclein is able to undergo conformational changes from random coil to β-sheet structure, needed to form fibrils. The study of Giasson et al. [28] indicates that the region between amino acids 71 and 82 is necessary for the polymerization of this protein into filaments. Moreover, this middle region is suggested to favour intermolecular hydrophobic interactions promoting aggregation [28], while ends comprise a membrane-binding domain [8,27].

In this study, the aggregation of α-synuclein was evaluated by ultra-structural analysis in the presence of three different surfactants, below and above their CMC. The choice of ionic surfactants was based on the micelles shape. SDS and CTAC both form spherical micelles of approximately the same size. The non-charged surfactant OG was chosen because of its purity in comparison with other non-charged surfactants (Table 2).

The CMC values for the surfactants in phosphate buffered saline (PBS) with and without α-synuclein were determined by surface tension measurements and are listed in Table 3. Below the CMC, the experimental data of surface tension follow the Langmuir–Szyszkowski equation [29]. Above the CMC, the surface tension is almost constant and is shown as a linear curve. The CMC values were determined by the interception of both curves (Supplementary Figure S1: example of one adjustment for condition) [30].

In charged surfactants, the CMC significantly decreased (*p* < 0.05) in the presence of the α-synuclein, presumably because of the fact that the protein screens the electrostatic repulsion between the head groups of SDS and CTAC. The effect is more pronounced for SDS.

The aggregation of α-synuclein alone and in the presence of surfactants was evaluated over 9 days. To accelerate the aggregation process, two glass beads per well were used. A longer experiment (350 h) was also performed without glass beads to see whether they influence the interaction of surfactant with α-synuclein. The fluorescence emission of Thioflavin T (ThT) in solution was low because the excitation energy was dissipated through rotation around the central axis in its molecular structure. However, in the presence of β-sheet fibrils, ThT molecules bound to the α-synuclein protein, consequently restricting the molecule rotation, and increasing the fluorescence emission [31,32,33,34]. Thus, the amount of fibril formation was directly proportional to the intensity of the fluorescence emission [35].

**Figure 2:**
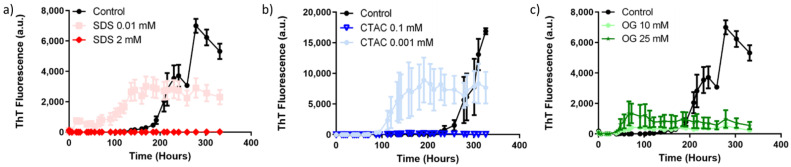
Aggregation kinetics of α-synuclein (35 µM) in the presence of buffer (control) or surfactant in phosphate buffer (pH 7.4 at 37 °C). (**a**) 0.01 and 2 mM of SDS; (**b**) 0.1 mM and 0.001 mM of CTAC; (**c**) 10 mM and 25 mM of OG. Differences in control curves for panels a–c and b are due to reactions being run in separate plates.

During the incubation period, it was possible to observe a significant increase (*p* < 0.05) in the fluorescence intensity, suggesting an intensification of ThT binding to α-synuclein alone (Figure 2), thus indicating the fibril formation. In addition, differences in the controls are due to each experiment being run in different plates, each one of them considering different control reactions. α-synuclein is known to display a fast elongation phase. In that sense, variability between different reactions is expected, as reported in previous works [33,34].

In the presence of the SDS concentration below the CMC (0.1 mM), an increase in the ThT fluorescence for α-synuclein was observed immediately after one day (Figure 2a and Table 4). This indicated that SDS monomers induced α-synuclein misfolding. The negatively charged monomers were supposed to neutralize the positive charge of α-synuclein, mainly present at the N-terminus. Here, the tails of SDS molecules are exposed, making the region more hydrophobic. Despite the electrostatic repulsions between the negative charges of α-synuclein molecules, hydrophobic interactions can still occur, thus promoting the protein polymerization.

The opposite effect is observed when the α-synuclein is in the presence of SDS above the CMC (2 mM) (Figure 2a and Table 4). Our results show that, at concentrations above the CMC, SDS inhibits the aggregation of α-synuclein. On the contrary, 0.01 mM SDS facilitated the misfolding of α-synuclein. Interestingly, maximum ThT fluorescence in the presence of SDS was different to the one generated in the absence of this detergent, suggesting the formation of different structures. After 14 days, no amyloid structures appear in the solution in the presence of 2 mM SDS. These results may be due to the action of negatively charged micelles, which can bind to the positively charged a.a. at the N-terminus. Owing to the structure of micelles (hydrophobic tails oriented to the core), only the negative charges of α-synuclein are exposed. As a result of this, only electrostatic repulsions between the negatively charged C-terminus of the protein occur, thus preventing the α-synuclein aggregation. These results are in agreement with a previous study showing the interaction of SDS micelles with α-synuclein using NMR [36]. This report suggests that the protein is positioned on the surface of the micelle and that the NAC-region is partially inserted in the micelle. This might be the reason inhibition of aggregation is observed at SDS concentrations above the CMC. If the NAC-region is not available, the hydrophobic interactions between the NAC-region and C-terminal would be reduced. Moreover, it would not be possible for the electrostatic interactions between the N- and C-terminal regions to occur. In that sense, the obtained structures would not be favoured to induce fibril formation.

With CTAC concentrations below the CMC, an increase in the fluorescence intensity was observed in the fifth day of incubation (Figure 2b and Table 4). This indicates that CTAC monomers bind with negatively charged a.a. by electrostatic interactions mostly present in the C-terminus, strongly favouring fibril formation by hydrophobic interactions. However, the time taken for α-synuclein to aggregate in the presence of CTAC was higher compared with SDS below the CMC. Above the CMC, the positively charged micelles interact with the negative C-terminal part of the protein. There, repulsion between positively charged micelles and the positively charged N-terminus a.a. should occur. Consequently, the hydrophobic parts of the C-terminus would be less accessible owing to the presence of the micelles. However, the surfactant tails are not exposed and the hydrophobic interactions between the NAC-region and protein’s C-terminus part of the protein would not be affected by the surfactant’s tails (Figure 2b). In agreement with these results, a study focusing on the influence of the pH on the α-synuclein structure [37] indicated that the aggregation is faster at low pH. The highly negative charged C-terminal domain of the protein would then be neutralized, and this region should become highly hydrophobic. The protons are more comparable with micelles than the monomers, because of the lack of the exposition of the hydrophobic tail.

Further experiments involving in vitro aggregation of α-synuclein in the presence of different concentrations of CTAC (Figure 2b) show that the presence of CTAC at the CMC (0.1 mM) partially inhibits protein aggregation. On the contrary, concentrations below the CMC (0.001 mM) substantially accelerate the misfolding and polymerization of this protein. As observed for the SDS protein aggregation assay, the different maximum fluorescence of α-synuclein aggregates in the presence or absence of detergents suggests the formation of different types of misfolded inclusions.

The non-ionic surfactant OG inhibited α-synuclein fibrillation at both concentrations below and above the CMC over 9 days (Figure 2c and Table 4). At concentrations below the CMC, the tails of OG monomers were expected to interact with the hydrophobic region of α-synuclein. This would cause less exposure of the hydrophobic surfaces of α-synuclein. In that scenario, only electrostatic interactions would be possible and long-range hydrophobic interactions should be prevented. Above the CMC, the OG micelles are formed around the protein. A potential scenario is that hydrophobic side chains of α-synuclein are positioned into the micelle core, by hydrophobic interactions with the OG tails. This model has been described for the interaction between the uncharged surfactant n-dodecyl-α-D-maltoside and the peptide co-poly-L-(lysine. phenylalanine) 1:1 HBr [38]. In that model, only the hydrophobic side chains might be available to interact. In the NAC-region, the amount of hydrophobic a.a. is high and only three polar a.a. are present (two glutamic acids at position 61 and 83, and one lysine at position 80). OG micelles are thought to interact mostly at the NAC-region. In this model, α-synuclein could be located just between the OG micellar head groups and the hydrocarbon chain core. Considering the hydrophobic side chains directed into the micelle and the polar side chains oriented out of the micelle, protein–micelle interaction would occur mostly at the NAC region, hindering hydrophobic interactions with the C-terminus. The OG micelles apparently reduce the availability of the hydrophobic a.a. when compared with OG monomers alone. This can explain the decrease in fluorescence intensity for protein in the presence of OG above the CMC.

In agreement with the previously presented data, in vitro aggregation of α-synuclein was inhibited in the presence of OG at concentrations above and below the CMC (Figure 2c).

**Figure 3:**
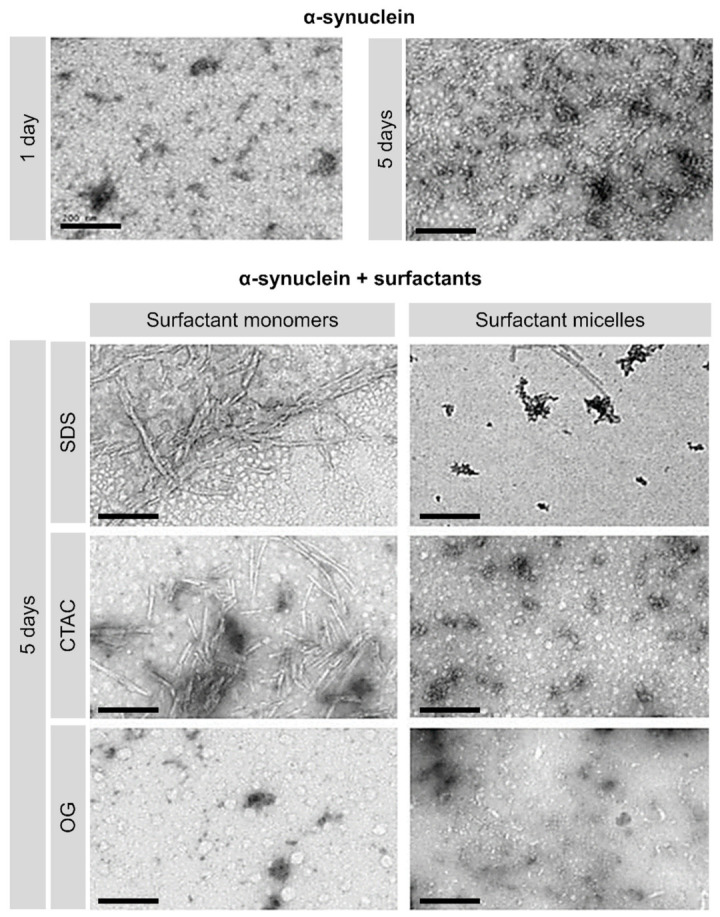
Transmission electron microscopy (TEM) images showing the effect of SDS, CTAC, and OG on α-synuclein aggregation. The α-synuclein concentration was 15 μM. The samples were incubated for 5 days at 37 °C in the presence or absence of surfactants in phosphate buffer, 137 mM NaCl, pH 7.4. The studied surfactant concentrations were below and above the critical micelle concentration (CMC) (CTAC 0.05 mM and 1 mM; SDS 0.1 mM and 2 mM; OG 10 mM and 40 mM) in phosphate buffer (pH 7.4). The scale bars correspond to 200 nm.

Ultra-structural analyses showed that both SDS and CTAC at concentrations below the CMC promote the aggregation of α-synuclein (Figure 3). These data support our observation in the protein aggregation assays. In these conditions, the surfactants led to the formation of fibrils, with a mean diameter of approximately 12 nm. The observed increase of approximately 1 nm in the fibrils size was caused by the attachment of the negative-staining agent (uranyl acetate). Furthermore, while SDS promoted the formation of long fibrils, ranging between approximately 200 and 400 nm, CTAC induced the formation of slightly shorter fibrils, with lengths ranging from 100 nm to 200 nm. The obtained results for α-synuclein fibril size are in agreement with the literature [39].

In the case of α-synuclein incubated with the charged surfactants at micellar concentrations (concentration above the CMC), only small aggregates corresponding to pre-fibrillar oligomers were observed, indicating that they inhibited α-synuclein fibrillogenesis. Images in this case were similar to the ones observed for the peptide in the initial state (Figure 3, time = 1 day).

TEM analysis of α-synuclein aggregation in the presence of the non-ionic surfactant OG, both sub-micellar and micellar concentrations prove the inhibitory effect of the surfactant when compared with the presence of SDS and CTAC micelles. While essentially long and thin fibrils and some protofibrils are present in the absence of surfactants, these structures are absent in the presence of OG. Instead, only short fragments corresponding to dimers, trimers, and oligomeric structures were observed.

**Figure 4:**
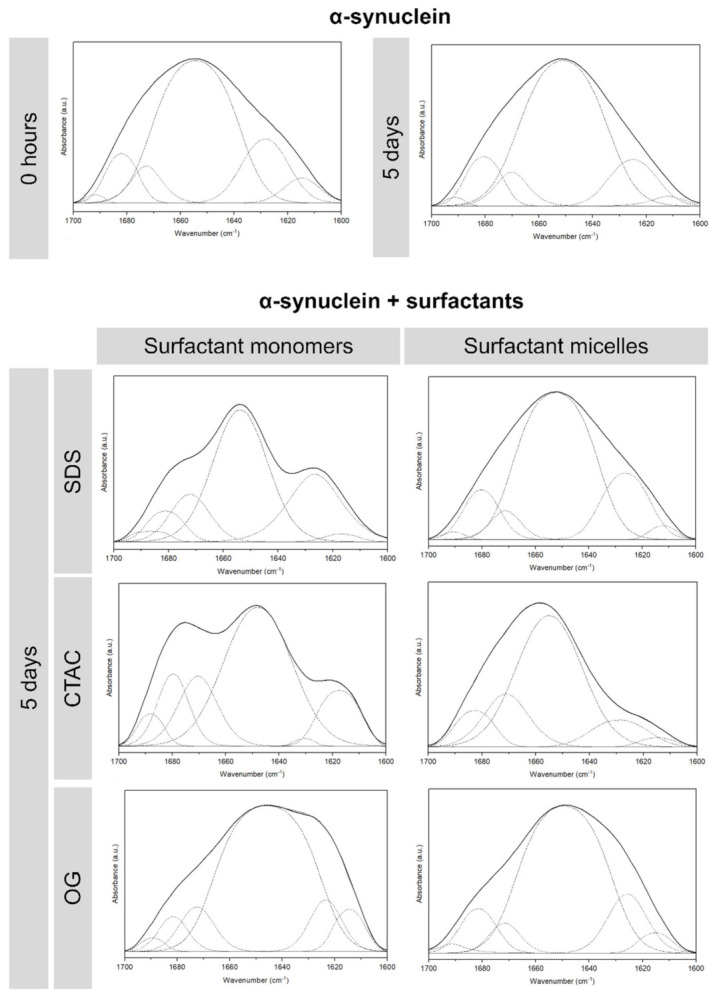
Fourier transform infrared spectroscopy (FTIR) spectra of α-synuclein (15 µM) in the presence of buffer (control) or surfactant in phosphate buffer (pH 7.4 at 37 °C): CTAC (0.05 and 1.00 mM), SDS (0.10 and 2.00 mM), or OG (10.00 and 40.00 mM). The dashed lines represent the curve-fitted components for secondary structure analysis and the solid lines represent the FTIR spectra.

The conformational changes of α-synuclein in the presence of SDS, CTAC, and OG monomers and micelles was also evaluated by ATR-FTIR spectroscopy. Figure 4 presents the FTIR spectra of the amide I region (1700–1600 cm^−1^) for this protein in the different conditions tested. The deconvoluted FTIR spectra of proteins in band 1700–1600 cm^−1^ are associated with the C=O stretching vibration of the peptide bonds [40]. For α-synuclein in the absence of surfactants, a main peak at around 1650 cm^−1^ is observed, which is typical of unfolded polypeptides [41]. As expected, for α-synuclein in the presence of SDS and CTAC monomers after 5 days of incubation at 37 °C, a decrease in the peak located at 1650 cm^−1^ was observed. In the presence of SDS monomers, a decrease of around 25% was observed. In addition, CTAC monomers induced a decrease of this feature in around 20%. Moreover, the α-synuclein solution containing CTAC monomers showed an augmented peak (approximately 79%) between 1685 cm^−1^ and 1695 cm^−1^, which is characteristic of antiparallel β-sheet structure [42]. In the case of α-synuclein solution containing SDS monomers, the peak intensity substantially increased in the band around 1617 cm^−1^, which is related to β-stands in aggregated structures. This is indicative of inter-molecular β-sheet conformation with strong hydrogen bonds. Moreover, the peak between 1620 cm^−1^ and 1638 cm^−1^, which is also characteristic of antiparallel β-sheet structure, is higher compared with the α-synuclein in buffer [42]. In contrast, this band frequency is low for the α-synuclein solutions containing OG monomers and micelles.

## 3. Materials and Methods

### 3.1. Material

α-synuclein (Sigma-Aldrich, Steinheim, Germany) monomers were dissolved in phosphate buffer (50 mM) containing Na_2_HPO_4_ and NaH_2_PO_4_ (Sigma-Aldrich, Steinheim, Germany) at 315 µM concentration in order to obtain the stock solution.

Stock solutions of surfactants were prepared by dissolving SDS (≥98.5%, MW 288.38, Sigma-Aldrich, Steinheim, Germany), CTAC (25 wt% solution in water, MW 320.01, Sigma-Aldrich, Steinheim, Germany), or OG (≥98%, MW 292.37, Sigma-Aldrich, Steinheim, Germany) in phosphate buffered saline (PBS), pH 7.4 (10 mM phosphate buffer, 2.7 mM potassium chloride, and 137 mM sodium chloride, Sigma-Aldrich, Steinheim, Germany).

### 3.2. Measurement of the Critical Micelle Concentration

The CMC of the surfactants was determined using surface tension measurements at 21 °C. Serial dilutions of the surfactants were prepared with an increasing surfactant concentration, in the absence or presence of 25 µM α-synuclein at final concentration. The hanging drop method was selected for the measurements of surface tension [24] using an OCA 15 plus optical contact angle system (DataPhysics Instruments GmbH, Filderstandt, Germany). 

### 3.3. Transmission Electron Microscopy

α-synuclein at a final concentration of 15 µM was incubated for 5 days with CTAC (0.05 and 1.00 mM), SDS (0.10 and 2.00 mM), or OG (10.00 and 40.00 mM) at 37 °C in 10 mM PBS buffer (pH 7.4). For the protein visualization, 5 µL of each sample was placed on carbon-formvar coated 400 mesh spacing grids and left to adsorb for 5 min. The samples were negatively stained with 2% (*w*/*v*) uranyl acetate for 45 s and visualized at an accelerating voltage of 80 kV (Jeol JEM 1400, Tokyo, Japan) [43].

### 3.4. Thioflavin T Binding Assay

To prepare the Thioflavin T (ThT) (MW 318.86, Sigma-Aldrich, Steinheim, Germany) stock solution, 8.0 mg of ThT was added to 10 mL of PBS [44]. This solution was then filtered through a 0.2 μm syringe filter. Before each measurement, a ThT working solution was prepared diluting the ThT working solution (1 mL of ThT stock solution in 50 mL of PBS, 50 µM).

For the fluorescence measurements, 17.5 µL of each sample was mixed with 60 µL of ThT working solution in an untreated 96-well plate (NUNC, black polystyrene, flat bottom) (α-synuclein at final concentration of 15 µM with or without surfactant). At each well, two glass beads with a diameter of 1.25–1.40 mm were added to accelerate the aggregation process. The fluorescence intensity was obtained using a Biotek Synergy (Winooski, Vermont, USA) fluorescence spectrometer after stirring for 30 s at 37 °C every 20 min for 5 days. The assay was performed with excitation and emission filters of 420/50 and 485/20 nm, respectively. The fluorescence spectra were corrected using appropriate controls (solutions without α-synuclein).

### 3.5. α-Synuclein Aggregation Assay

The α-synuclein aggregation assay was performed in the presence of surfactants at concentrations below or above their CMC. Briefly, α-synuclein was purified from bacteria as described in Shahnawaz et al. [45]. The monomeric stage and purity of this protein preparation were confirmed by the use of 100 kDa cut-off filters (Amicon Ultra, Millipore, Burlington, MA, USA) and silver staining. Recombinant α-synuclein at a concentration of 35 µM was incubated in 100 mM piperazine-N,N’-bis(ethanesulfonic acid), pH 6.5, 500 mM NaCl, and 5 µM ThT in untreated 96-well plates that were inert tothe protein aggregation components. Surfactants were added to the reaction at different concentrations (Figure 2), with a final reaction volume of 200 μL. Samples were subjected to cycles of incubation/agitation (1 min at 500 rpm followed by 29 min without shaking) at 37 °C. Aggregation of α-synuclein was monitored by measuring ThT fluorescence emission at 485 nm after excitation at 435 nm using a microplate spectrofluorometer (Gemini EM; Molecular Devices, San Jose, CA, USA). ThT fluorescence data were also obtained for the controls—surfactants with ThT. These values were present at background levels and did not significantly change over time. The controls were subtracted from their corresponding samples to generate the final plots.

Protein/surfactant reactions were compared to aggregation assays in the absence of surfactants. These controls were independently placed in each reaction plate and linked to their respective surfactant reaction.

### 3.6. Attenuated Total Reflectance–Fourier Transform Infrared Spectroscopy

The attenuated total reflectance–Fourier transform infrared spectroscopy (ATR–FTIR) spectra of α-synuclein solutions were obtained using a Bruker Alpha-P spectrophotometer, Germany. α-synuclein at a final concentration of 15 µM was incubated for 5 days with CTAC (0.05 and 1.00 mM), SDS (0.10 and 2.00 mM), or OG (10.00 and 40.00 mM) at 37 °C in 10 mM PBS buffer (pH 7.4) under medium agitation-inducing conditions. Then, 5 µL of each sample was applied on the surface of the FTIR crystal and dried using a nitrogen stream flow to form a thin film. The spectra were recorded in the amide I region from 1700 to 1600 cm^−1^ and background and controls with surfactants subtractions were achieved. Second derivative, Fourier self-deconvolution of the Amide I region, and curve fitting using Gaussian/Lorentzian functions were performed using the software OriginPro 2020b (OriginLab Corp, Northampton, MA, USA). 

### 3.7. Statistical Analysis

Results are presented as means ± standard deviation, from at least three independent experiments. Statistical analyses were performed using Student’s *t*-test or one-way analysis of variance. *p*-values < 0.05 were considered to be significant.

## 4. Conclusions

The aggregation mechanism of α-synuclein is still debatable because studies performed under different conditions lead to different proposed mechanisms. The fast aggregation kinetics and the different aggregation stages of protein samples are obstacles for uniform and generalized studies. The use of surfactants may contribute to a better understanding of the protein aggregation mechanisms associated with synucleopathies, including PD.

Here, we observed that α-synuclein decreases the CMC of charged surfactants. Surfactant monomers undergo electrostatic and hydrophobic interactions with the protein. This brings the surfactant monomers together, forming micelles in a faster way.

**Figure 5:**
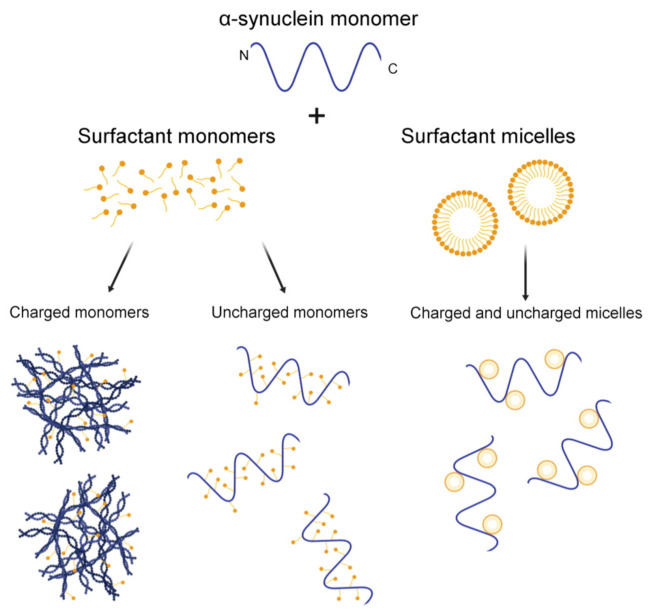
Schematic representation of the possible mechanisms for the interactions between α-synuclein with charged and uncharged surfactants at monomeric and micellar concentrations. The experimental data in this work show a significant induction of α-synuclein aggregation with charged monomers.

Our data show an induction of α-synuclein aggregation in the presence of negatively charged (SDS) and positively charged (CTAC) surfactants. This indicates that the SDS and CTAC monomers induce structural changes that favour protein misfolding and aggregation (Figure 5). A reduction in the electrostatic repulsion between the N- and C-terminal regions of the protein, and the hydrophobic interactions between the NAC-region and the C-terminus, seems to be critical to undergo aggregation.

## Figures and Tables

**Table 1:** Numeric overview of the charged and hydrophobic a.a. of α-synuclein.

Region	N-Terminal	NAC ^1^	C-Terminal	α-Synuclein
Total number of a.a.	60	35	45	140
Number of charged a.a.	18	3	18	39
% Charged	30.0	8.6	40.0	27.8
Total charge	+4	−1	−12	−9
Number of hydrophobic a.a.	28	20	16	64
% Hydrophobic a.a.	46.7	57.1	35.6	45.7

^1^ NAC (non-amyloid beta component).

**Table 2:** Structures of the surfactants cetyltrimethylammonium chloride (CTAC), sodium dodecylsulfate (SDS), and octyl β-D-glucopyranoside (OG).

Surfactant	SDS	CTAC	OG
Charge	Anionic	Cationic	Nonionic
Molecular weight (Da)	288.38	320.01	292.37
Structure	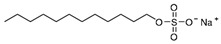	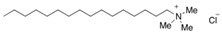	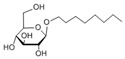
Shape of the micelle	Spherical	Spherical	Cylinder
Size of the micelle (nm)	4.8	4.8	R:1.3
			L: 9.6

**Table 3:** Critical micelle concentration (CMC) values from the surface tension measurements of SDS, CTAC, and OG in the absence or presence of α-synuclein (25 µM) in phosphate buffer (pH 7.4) at 21 °C (*n* = 3).

Surfactant	CMC without α-Synuclein (mM)	CMC with α-Synuclein (mM)
SDS	1.27 ± 0.04	0.42 ± 0.01
CTAC	0.17 ± 0.03	0.12 ± 0.04
OG	19.9 ± 0.3	22.5 ± 0.9

**Table 4:** Evaluation of α-synuclein aggregation by the thioflavin T (ThT) assay in solutions of 0.1 mM and 2 mM SDS, 0.05 mM and 1 mM CTAC, and 10 mM and 40 mM OG in phosphate buffer (pH 7.4 at 37 °C). “+” means more aggregation than α-synuclein alone and “-” means less aggregation than α-synuclein alone.

	α-Synuclein in SDS Solutions			α-Synuclein in CTAC Solutions			α-Synuclein in OG Solutions		
Days	1	5	9	1	5	9	1	5	9
Above CMC	-	-	-	-	-	-	-	-	-
Below CMC	+	+	+	-	+	+	-	-	-

## Supplementary Materials

The following are available online at https://www.mdpi.com/article/10.3390/ijms222212509/s1.
