# Supplementary material for: (De)stabilization of Alpha-Synuclein Fibrillary Aggregation by Charged and Uncharged Surfactants
Source: Int J Mol Sci. 2021 Nov 19;22(22):12509. doi: 10.3390/ijms222212509 (PMC8624236; doi:10.3390/ijms222212509)
Supplement: Supplementary file 1 [file ijms-22-12509-s001.zip › ijms-1444026-supplementary.pdf]

Supporting Information

(De)stabilization of alpha-synuclein fibrillary aggregation by charged and uncharged surfactants

Joana A. Loureiro, Stéphanie Andrade, Lies Goderis, Ruben Gomez-Gutierrez, Claudio Soto, Rodrigo Morales, Maria Carmo Pereira

Figure S1: Surface tension of in function of the surfactant concentrations of sodium dodecylsulfate (SDS), cetyltrimethylammonium chloride (CTAC) and octyl β-D-glucopyranoside (OG) in the absence or presence of α-synuclein (25 µM) in phosphate buffer (pH 7.4) at 21 ºC.

<table>
<tr><th></th><th>Absence of α-synuclein</th><th>Presence of α-synuclein</th></tr>
<tr><td>SDS</td><td>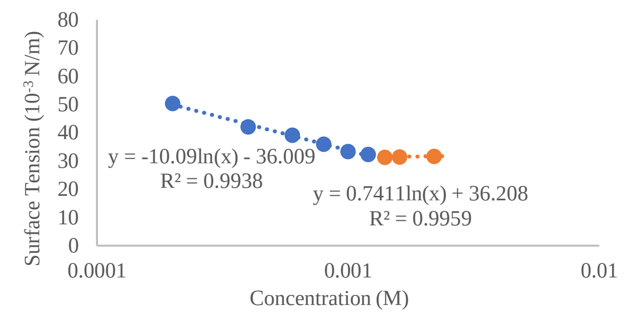
</td><td>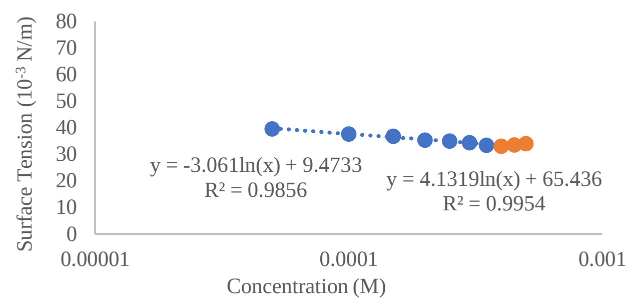
</td></tr>
<tr><td>CTAC</td><td>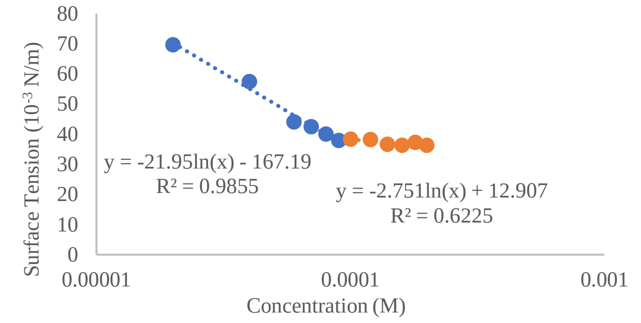
</td><td>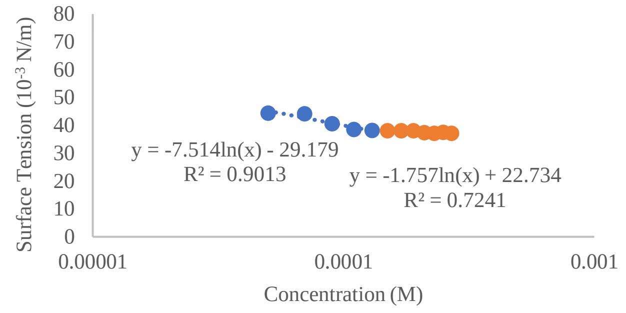
</td></tr>
<tr><td>OG</td><td>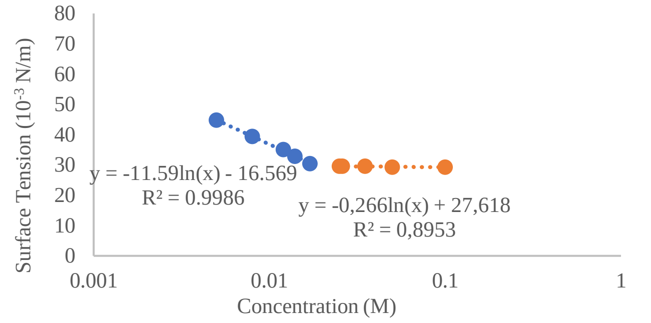
</td><td>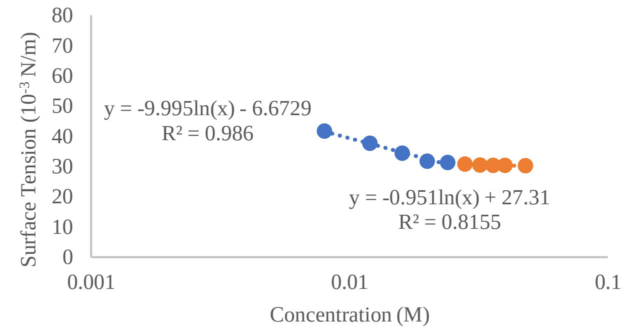
</td></tr>
</table>
